# Some Account of the Epidemic of Scarlatina Which Prevailed in Dublin from 1834 to 1842 Inclusive, with Observations

**Published:** 1844-01

**Authors:** 


### ART. XV.

1. *Some Account of the Epidemic of Scarlatina which prevailed in Dublin from* 1834 *to* 1842 *inclusive, with Observations.* By HENRY KENNEDY, A.B. M.B. T.C.D. &c., one of the Medical Officers of St. Thomas's Dispensary.—*Dublin*, 1843. 8vo, pp. 214.

2. *Scarlatina, and its Treatment on Homœopathic Principles.* By JOS. BELLUOMINI, M.D.—*London*, 1843. 8vo, pp. 32.

3. *Untersuchungen und Erfahrungen über das Kohlensaure Ammonium und seine Heilkräfte gegen das Scharlachfieber, &c.* Von A. W. BODENIUS.—*Heidelberg*, 1842. 8vo, pp. 170.

*Experimental Researches into the powers of the Carbonate of Ammonia as a Therapeutical Agent in the Treatment of Scarlatina.* By Dr. A. W. BODENIUS.

FEW tasks are more interesting than that of tracing the various changes which diseases have undergone in the course of time. Some maladies, once the scourges of our race, have ceased to exist, or have assumed so mild a form as no longer to excite alarm; while others, which in bygone days were comparatively trivial, are now dreaded as dangerous or fatal. This fact did not escape the notice of the earlier medical writers. "Mutantur quidem morbi," says Van Helmont, "larvantur, augentur, degenerant. Nec veteres morbi respondent amplius descriptionibus avorum. Postremo nuper ad nos venerunt morbi novi, et antiqui deinceps vix amplius respondent ad nomina et descriptiones avorum, quia signa et proprietates alienas induerunt, quibus larvati incedunt." No disease has afforded more striking illustrations of such mutations than scarlatina has done, since the time when it first ravaged Spain and Italy under the name of the *Garrotillo.* On these, however, we cannot now dwell. Our present purpose only allows us to observe, that about the commencement of the present century, scarlet fever assumed an unwonted malignancy, and spread epidemically, especially in Germany, where its ravages were frightfully severe. Thus, in the neighbourhood of Wittenberg,* four hundred persons were attacked in the course of fourteen days, and one in ten died. In London, too, the disease ran a rapidly fatal course, and was distinguished by the severity of the sore throat by which it was attended. It prevailed also in Ireland as an epidemic disease from the year 1801 to 1804, running a fatal course in many instances, and presenting much of the character of scarlatina maligna. It then changed its character, and continued for many years so mild, that Dr. Graves informs us in his lectures, "although scarlatina epidemics recurred very frequently during the next twenty-seven years, yet it was always in the simple or mild form, so that I have known an instance where not a single death occurred among eighty boys attacked in a public institution." With that self-complacency, so natural to mankind, this diminished mortality was attributed to the altered plan of treatment, to the doctrines of Brown having been discarded, and antiphlogistic means resorted to in place of the stimulating and exciting method which he advocated. In 1834, however, the disease resumed the grave featnres it seemed to have altogether lost, and Dr. Graves states that the Irish physicians of the present day are

* Schnurrer, Chronik der Seuchen, ii Band, s. 437.

forced to confess that in spite of their boasted improvements they have not been more successful in 1834-5 than their predecessors were in 1801-2.

It appears from the account given by Dr. Graves, that scarlatina in Dublin was seldom attended with danger until the year 1831, when a remarkable alteration began to be observed in its character; its previous inflammatory type was replaced by a concealed insidious form of fever, attended with great debility. It now, too, often terminated fatally, and began to extend much more rapidly and universally than before. Neither the state of the weather, nor the abode of the patient, seemed to have any influence in modifying its character or diminishing its prevalence. It is to a description of this epidemic that Dr. Kennedy's little work is devoted. Unfortunately, he furnishes us with no account of its rise, progress, and decline, or of the various fluctuations in character which it displayed at different times, or of how it seemed to be modified by the prevalence of other diseases, though such points should always be related by the historian of an epidemic. He does not attempt any classification of the various forms which the disease assumed; if we except a division he makes when describing the post-mortem appearances into the simply malignant, and the complicated; the latter comprising those cases in which there was very serious affection of the throat. Neither does he give any description of the disease as a whole, but treats of each symptom separately, and then details a number of cases which occupy sixty pages, and from these *membra disjecta*, the reader is left to form a picture of the disease as he best may. The faults of the book, too, are not confined to defects in arrangement, but the style is obscure and awkward in the extreme. It is, we think, no unreasonable requirement to expect that a gentleman who writes A.B. after his name should be able to express himself in English, but Dr. Kennedy breaks the rules of syntax in nearly every page, and we have often been able to gather the meaning of a sentence only by carefully comparing it with the context.

We will now endeavour to furnish our readers with a sketch of the main features of the disease as we can collect them from Dr. Kennedy's account, which, in spite of all its defects, is evidently the work of an honest, diligent, and truthful observer. In the simply malignant form, the eruption usually faded much after death; but in some cases in which it had not appeared until late in the disease, it went on rapidly increasing until death, "and continued to do so for a considerable time after, till at last the body in many parts became black, and taken as a whole was of a very dark colour. In these cases great swelling took place after death, and the signs of decomposition set in very early." (p. 3.) Vibices and petechiæ were present it many instances, and a lividity of the extremities as great as in patients who have died of cholera. In many of those parts too, where the body had been exposed to pressure, the integuments were found in a state of slough, which, however, seldom extended for any great distance. The cerebral substance was usually injected, and the ventricles contained fluid. Sometimes, too, there was an appearance of extravasation of blood beneath the arachnoid, such as is occasionally met with in typhus fever. Usually the appearances found in the brain after death, were in exact proportion to the severity of the cerebral symptoms during the lifetime of the patient. The lungs were found highly congested, and often broke down very easily; the bronchi were loaded with frothy serum and their mucous membrane was much congested. The heart was loaded with black blood, petechiæ were sometimes present on its surface, and its texture was occasionally softened. The blood was usually thin and watery. Congestion in patches, of the different abdominal viscera, was all that was found even in cases where during life the abdominal symptoms had been most striking.

The second, or complicated malignant form of the disease, was characterized by the severity of the affection of the throat, which sometimes came on when the fever was at its height, but quite as often after convalescence had begun. It generally proceeded very rapidly, and usually affected both sides of the neck, rendering the integuments extremely hard, sometimes reaching down to the pectoral muscle, and attended with great swelling, which subsided very rapidly after death. Serum or pus was found infiltrated into the cellular tissue; sometimes the pus was collected into one large abscess, or formed a number of little ones. In all these cases there was a great tendency to sloughing; and when that took place the lymphatic glands sometimes suppurated, and even the upper part of the sterno-mastoid muscle became disorganized. In three instances of this kind the patients bled to death, owing to the vessels of the neck giving way, and in two of these cases the jugular vein was ascertained to have been the source of the hemorrhage. There was another form of swelling about the neck, caused by the effusion of lymph only, which attained a large size very rapidly, was attended with no discoloration of the skin, but with extreme hardness of the integuments, and showed no disposition to suppurate. It does not appear that sloughing of the mucous membrane of the throat was the cause of death in any instance, but diphtheritis seems not to have been an unusual complication, and œdema of the glottis proved fatal in some instances.

"Both of these forms of disease were found accompanied by ulcerations varying in extent and number,—thus there was very constantly one in the upper part of either tonsil; its edges were irregular, and its depth usually very considerable. I also found ulcers in a very distinct form about the chordæ vocales; here, also, they were deep, but of a more circular form, and about the size of a grain of large shot; in one instance the alæ of the thyroid cartilage had become diseased." (p. 18.)

These ulcerations of the mucous membrane of the larynx seem somewhat analogous to those described by Dr. West as occurring in the course of measles.

Among the sequelæ of the disease Dr. Kennedy notices some forms of wry-neck, which he believes take their origin from the affection of the throat and neck we have just described. He distinguishes three varieties of it, and speaks first of

"...... cases in which, when the patient was to a certain degree convalescent, it was found that the child's neck had become crooked, and that when any attempt was made to bring the head straight, it caused severe pain, referred to the upper part of the neck, and commonly to one side. The pathology of this form of wry-neck is yet to be made out. Mr. O'Ferrall suggests that the inflammation of the throat spreads to the neighbouring textures, so as to engage those of the spine, and sometimes in this way causes caries. That this might occur is highly probable, but it must not be forgotten that apparently well-marked disease of this part of the spine may be with, or without any serious lesion, and in proof of it I would refer to what Dupuytren has published on the subject. There are, however, two other affections, of the nature of which I have been able to satisfy myself, both of which cause deformity, and which it is very necessary to distinguish from what has been spoken of above, as they require a different form of treatment. The first has been alluded to before, when speaking of those swellings of the neck where nothing but lymph was effused; when, however, it causes wry-neck, its situation is then in the small muscles at the nape of the neck, and sometimes in the upper portion of the trapezius; some pain attends it when pressure is made, and it is a very obstinate affection to remove. I have seen the same form of disease after delivery, of which there was a well-marked example not long since, in Sir Patrick Dun's hospital, under the care of Dr. John Ferguson. The pathology of this form of wry-neck consists, as already stated, in the effusion of lymph, and the matting together of all the parts about the nucha. The third form is quite different from either of the preceding; it is a much more common affection; that is, it is met with after other affections besides scarlatina. Thus I have seen it after infantile remittent fever, measles, and also one case of burn where the neck itself was not injured. It is this form of disease which many have seen in connexion with certain cases of hysteria. It arises from a spastic state of the muscles of one side of the neck, more particularly engaging the sterno-mastoid, and seems to be caused by the patient lying in one position in bed for a considerable period. The last case I saw of it laboured under the anasarca of scarlatina at the same time. It also is a very obstinate affection." (pp. 19-20.)

Inflammation and suppuration of the internal ear, purulent effusion into some of the joints, or the formation of abscesses in the soft parts of the extremities, were other sequelæ of the disease. Pneumonia, too, was by no means unusual, and was but seldom associated with pleurisy; which statement, if quite correct, constitutes an exception to what is usually the case. The kidneys were generally quite healthy, and this even in cases where the presence of albumen in the urine during the lifetime of the patient might have led to the expectation that they would be found diseased. Sometimes, however, the kidneys were greatly congested, and thrice they presented the appearances characteristic of Bright's disease.

In the second chapter Dr. Kennedy describes the symptoms of the disease, which varied so much in different cases that he considered it would be impossible to arrange them under the usual heads of scarlatina simplex, anginosa, or maligna. He states, that many cases which began with the most alarming symptoms, ran a very mild course; while in other instances the opposite of this was observed. In default of any attempt at classification by Dr. Kennedy, we are glad to be able to borrow from Dr. Graves's account of the epidemic, who states that the severer cases assumed one of three forms. In those cases which he refers to the first form, besides fever, sore throat, and headach, there were violent congestion of the brain and determination of blood to the head, giving rise at a very early period to convulsions and apoplectic coma. The second form was marked, in addition to the ordinary symptoms of the disease, by a severe headach, which existed from its very commencement; by pain in the back, and very great irritability of the stomach and bowels. This irritable state of the abdominal viscera depended on cerebral congestion, and resembled that form which accompanies and sometimes masks the progress of acute hydrocephalus. Dr. Graves found this variety of scarlatina extremely dangerous and very little under the power of remedial agents. Some cases, too, put on the third form, and ran a very insidious course, closely resembling that observed in many instances by Dr. Withering in the epidemic which came under his notice. The patients advanced favorably, with comparatively mild symptoms, till the eighth or ninth day; when an exacerbation of fever occurred, with the return of sore throat, which speedily rendered deglutition difficult or almost impossible. Great swelling of the parotid and submaxillary glands now came on, and often surrounded the neck as with a collar; while a diphtheritic formation occurred in the mouth and extended into the pharynx. Fever, of a typhoid character, attended the local symptoms; and death took place after being preceded, for the most part, by some hours of distressing restlessness.

But to return to the description of the different symptoms by Dr. Kennedy. It appears that, in the greater number of cases, the attack of the disease was very sudden; sickness, vomiting, and dizziness occurring quite instantaneously in persons apparently in perfect health; or occasionally sore throat would come on just as suddenly. After these symptoms, which were generally attended with more or less marked pyrexia, had lasted for a few hours, a general condition of collapse, with great depression and phenomena like those of the cold stage of severe remittents came on, and lasted for from two to five hours, when reaction took place, and all the more prominent symptoms of the fever supervened. Among these symptoms were sore throat, which, in a more or less severe form, was never wanting; though by no means always attended with pain. In some cases, indeed, a very serious degree of sore throat was found to exist without the patient having made any complaint about it. In addition, too, to the more common form of sore throat, a variety was sometimes met with characterized by an aphthous condition of the mucous membrane; or, in other instances, the uvula was so infiltrated with serum as to acquire an enormous size. In those cases in which a false membrane existed on the tonsils, velum, or back of the throat, death took place more speedily than under other circumstances. Sloughy ulcerations of the tonsils were of very frequent occurrence, and were often attended with hemorrhage from the mouth, though never to such an extent as to prove fatal. Such ulcers often continued unhealed for many months after the subsidence of the other symptoms; a circumstance to which Dr. Kennedy attributes the long persistence, in many instances, of the contagious properties of scarlatina.

The date of the appearance of the eruption varied very much. In the great majority of cases, indeed, it showed itself within the first twenty-four hours; but sometimes an interval of two or three days elapsed between the occurrence of the first symptoms and the appearance of the eruption. It remained out from three to five days, and assumed a much darker colour before its disappearance than it presented at its outbreak. It was succeeded by desquamation; between the extent of which, however, and the extent and intensity of the eruption, there did not seem to be any connexion. The character of the eruption varied greatly; sometimes it was intermixed with an eruption of a miliary character; but the presence of that did not betoken anything serious. It was to be regarded as an occurrence of much graver import when a second crop of eruption appeared twenty-four hours after the first. It usually showed a greater intensity than the first eruption, but receded after having remained out only a few hours. The eruption varied also very much in the extent of surface that it affected, and sometimes it shifted rapidly from place to place, an irregularity always of evil omen.

The eye was affected in various ways, to which Dr. Kennedy attaches considerable importance in a semeiological respect. In cases where there was delirium, more or less injection of the sclerotica existed, which was very different from the ordinary suffusion of the eye met with in typhus fever. It was unattended with intolerance of light, but was a bad sign which, in fatal cases, went on increasing until death. In most of these cases the pupil was contracted; but another condition of it was by no means unusual, that of perpetual oscillation wholly independent of the quantity of light falling on it, "the iris, as it were, gave the impression that it was in a state of nervous tremor, sharing in this respect with the nervous system in the same patient." This also was a bad sign.

The danger of the case was usually proportioned to the rapidity of the pulse. During the period of collapse, the pulse was usually weak and indistinct; rising in the course of eight or twelve hours to 120 or 130, and gaining in fulness. It then grew gradually weaker till about the fourth day, when, if the patients did well, it usually increased in tone and vigour, and diminished in frequency. Extreme feebleness of pulse was a very bad sign, and an attendant on the worst cases.

We pass over the sixty pages occupied with the detail of cases, and come to Dr. Kennedy's remarks on the treatment of the affection. He usually began with the administration of an emetic, but on account of the tendency to diarrhea, did not employ either antimony or ipecacuanha for this purpose, but mustard mixed with water. In cases, too, when there was a very high degree of fever and incessant restlessness, he was accustomed to repeat the vomit, and with very good effect, for he has seen its employment followed by general calming of the system, and even by sleep. Cold and tepid ablutions, and cold affusion to the head were frequently practised, the former moderating the heat of skin, the latter checking some of the more violent forms of maniacal excitement, which were of by no means of rare occurrence. General bleeding was hardly ever admissible, and even local depletion required to be practised with much caution. Stimulants were very often employed, but there was nothing peculiar either in these he selected, or in his manner of adminstering them. He tried opium, though not very extensively, in three classes of cases. The first class included those instances in which typhoid symptoms appeared at an early period in the disease, and were associated with signs of great depression and weakness. Here, a full opiate at bed-time was often extremely useful, particularly in those cases in which delirium continued unabated after the other symptoms had begun to decline. Cases of the second class resembled those instances of typhus fever in which Dr. Graves has been accustomed to administer tartar emetic and opium. They were distinguished by the prominence of the head symptoms, by their early occurrence, and their not being at all mitigated by the full appearance of the eruption. Opium was very useful in some of these cases; on others, apparently, it did not exert the slightest influence. It did not effect more than a very temporary good in cases of the third class; those, namely, in which diarrhea came on in the progress of the disease.

Anasarca is the only one of the different sequelæ of the affection of which Dr. Kennedy has treated at all at length. He met with it much more frequently during the latter than the earlier part of the epidemic.

It usually appeared about the twelfth day of the disease, though occasionally it showed itself sooner, and still oftener at a later period. In those cases where it occurred, the pulse continued quick after the disappearance of the eruption, slight fever was present, and desquamation did not take place. After some days, the child would begin to complain of nausea, with vomiting occurring occasionally for two or three days, and headach of an intermittent character, with which a peculiar sluggishness of the pupils was usually associated. This state of the pupils, too, was very remarkable in some cases where a post-mortem examination failed to detect any morbid condition of the brain. After these symptoms had lasted for some days, the dropsy would begin to make its appearance, sometimes very gradually, the extremities or face being puffy on one day, and of their natural size on the day following; at other times the anasarca came on very suddenly. In one instance it proved fatal in three days, in another seven weeks elapsed from the appearance of the dropsy to the supervention of the first alarming symptoms. In those cases which assumed a serious character, either head symptoms came on, or the chest became affected, or lastly, the patient died without any organ being specially involved. The premonitory head symptoms were much the same with those of ordinary hydrocephalus, but a dilated condition of the pupils came on at a very early period, and coexisted with distinct vision. The breathing was irregular, and the pulse would often fall twenty or thirty beats, and rise again to its former frequency of about 120 in the course of forty-eight hours; always, however, becoming slow when coma or convulsions were about to occur. The cases in which death was ushered in by chest symptoms did not present anything very striking. In cases of the third class, the patients sank under the violence of the febrile symptoms, without special affection of any organ in particular.

With reference to the condition of the urine, Dr. Kennedy's remarks are extremely meager and unsatisfactory. He states that albumen was not always present, but the only test he used for it was heat; and we have no information as to its specific gravity in any case. The state of the blood too, the presence of urea in it, or in any of the effused fluids, are points which, despite their great importance, Dr. Kennedy does not appear to have investigated.

The treatment he adopted was strictly antiphlogistic, and included the free use of purgatives, and general or local depletion whenever any cerebral or thoracic complication existed.

The work which stands second at the head of this article, is a popular exposition of the symptoms of scarlatina, and of its homœopathic treatment, and contains nothing particularly novel or interesting. The only point indeed which we shall notice is the old recommendation of belladonna and aconite, as remedies and prophylactics of the disease. "According to the principle of homœopathy, *similia similibus curantur*, which means that diseases are cured by remedies capable of producing in a healthy body similar maladies, belladonna ought to be, and is the specific remedy for scarlatina." (Belluomini, p. 19.) Now here we meet with the fallacy that runs through the whole of the homœopathic theory. Similarity of symptoms, not similarity of diseases lies, as Schultz justly remarks, at the base of all their therapeutic proceedings, and if they had not taken refuge in a Latin axiom, which has amply served the purpose of mystification, the so-called *scientific* theory of Hahnemann, would long since have been seen to deserve that character as little as did the old doctrine of signatures. The results obtained by the use of belladonna as a prophylactic are singularly conflicting; many of those who tried it and conceived that its use was attended by success, employed it in large doses; and M. Stievenant* of Valenciennes, whose paper on this subject was recently read before the Academy of Medicine in Paris, states expressly that the employment of belladonna in homœopathic doses did not give rise to any symptoms resembling those of scarlatina anginosa. M. Godelle has recently published an exceedingly prolix essay on scarlatina,† in which he labours to prove that belladonna is a prophylactic, but that its action, unlike that of vaccination, which destroys the liability to smallpox, is confined to removing the susceptibility to scarlatina during the time that it is employed, and ceases on its use being discontinued. Should this statement be substantiated, it may, perhaps, be the case that the narcotic power of belladonna, and of the kindred remedy aconite, blunts for the time the susceptibility of the constitution. On this supposition we might account for some who have been exposed to the contagion not contracting the disease. We confess that M. Guersant's favorable opinion of the prophylactic virtues of belladonna, make us hesitate in the expression of our disbelief in its powers. Still, it should be borne in mind, that the liability to scarlatina is much less than to smallpox and measles; that many persons pass through life without being affected by it: and that the general result of the more recent experiments with the drug were so much less favorable than those which were at first obtained, that Hahnemann was compelled to admit its frequent failures, and to resort to the supposition of a change in the fever, in order to explain the fact.‡ For those cases in which belladonna was not likely to be serviceable, Hahnemann suggested the use of aconite, in which he is followed by Dr. Belluomini and the rest of his disciples. We are not aware that aconite has been by any means so extensively tried as belladonna. Dr. Belluomini appears to believe, that with the two together, we might soon put an end to scarlatina in all its forms.

"Belladonna is only a preservative against smooth scarlatina, and therefore, it remains without effect when the prevailing epidemic is miliary scarlatina. Against this the preservative is aconite. Now as these two epidemics reign almost always at present promiscuously, whenever prudence suggests the use of a preservative, aconite and belladonna should be administered alternately. The doses of these medicines ought to be the same as those indicated for the cure, but taken at long intervals, beginning with aconite when miliary scarlatina is dreaded, and with belladonna if it be smooth scarlatina, and continuing the use of these medicines alternately. To aconite one day is the time which should be allowed for completing its action; to belladonna from three to six days should be given. In robust and active persons who perspire much, the action of belladonna will be extinct in the course of about three days; in persons in different conditions, its action may continue for six or seven days." (p. 27.)

* Bulletin de l'Académie Royale de Médecine, 15 Fevrier, 1843.

† Revue Médicale, Janvier, Mars, et Avril, 1843.

‡ Those who wish to see a brief summary of the various experiments on the prophylactic powers of belladonna will find it in Meissner's Forschungen, bd. iii, s. 302, and bd. vi, s. 441.

Dr. Bodenius's very wordy pamphlet may be divided into two parts, the first of which contains a report on the employment of the carbonate of ammonia, in an epidemic of scarlet fever that prevailed in the neighbourhood of Bretten, in the duchy of Baden, during the years 1837-8. He is so convinced of the specific virtues of this remedy, as to hazard the bold assertion that the "carbonate of ammonia will acquire the same importance as a remedy for scarlet fever, as is attached to vaccination as a preservative from smallpox." (p. 9.) The epidemic presented both forms of the disease, namely, the simple smooth scarlatina, and also the miliary variety. Both were characterized by severe affection of the throat, and swelling of the parotid glands occurred in three fourths of the cases. In addition to strict attention to the ordinary dietetic rules, it was his custom to employ the following mixture from the very outset of the disease.

| | | |
|---|---|---|
| ℞ Ammon. carbon. | . . | ʒss—ʒj. |
| Aq. destill. | . . . | ℥iij. |
| Syr. Altheæ | . . | ℥j. M. |

Of this mixture one or two teaspoonfuls were given every two hours, and continued, by night as well as by day, through the whole course of the fever, until the period of desquamation set in, when it was given only every four hours. Its action, when employed at the commencement of any case, was to quell the very troublesome vomiting which attended the outset of scarlatina, and afterwards to induce increased and as he considered, critical secretion from the kidneys.

The second and by far the larger part of the pamphlet contains a clumsily executed *résumé* of the opinions of different writers on scarlatina, and especially of the fluctuations that the carbonate of ammonia has undergone in the esteem of the profession. We call attention to the subject because we are convinced, from tolerably extensive experience, that though the remedy may not merit all the eulogies of Dr. Bodenius, it is nevertheless one of very great value in the treatment of scarlatina; that it deserves to be looked on in quite a different light from other stimulants, that it may be given advantageously under circumstances when the employment of other stimulants would be improper, and may often be used with benefit from the very commencement of the disease.

---

